# Smart Sensors and Smart Data for Precision Agriculture: A Review

**DOI:** 10.3390/s24082647

**Published:** 2024-04-21

**Authors:** Abdellatif Soussi, Enrico Zero, Roberto Sacile, Daniele Trinchero, Marco Fossa

**Affiliations:** 1 Department of Informatics, Bioengineering, Robotics and Systems Engineering (DIBRIS), University of Genoa, 16145 Genova, Italy; enrico.zero@dibris.unige.it (E.Z.); roberto.sacile@unige.it (R.S.); 2 iXem Labs, Department of Electronics and Telecommunications, Politecnico di Torino, 10129 Turin, Italy; daniele.trinchero@polito.it; 3 Department Mechanical, Energy, Management and Transportation Engineering, University of Genoa, 16145 Genova, Italy; marco.fossa@unige.it

**Keywords:** smart sensors, precision agriculture, smart data, optimization and control

## Abstract

Precision agriculture, driven by the convergence of smart sensors and advanced technologies, has emerged as a transformative force in modern farming practices. The present review synthesizes insights from a multitude of research papers, exploring the dynamic landscape of precision agriculture. The main focus is on the integration of smart sensors, coupled with technologies such as the Internet of Things (IoT), big data analytics, and Artificial Intelligence (AI). This analysis is set in the context of optimizing crop management, using resources wisely, and promoting sustainability in the agricultural sector. This review aims to provide an in-depth understanding of emerging trends and key developments in the field of precision agriculture. By highlighting the benefits of integrating smart sensors and innovative technologies, it aspires to enlighten farming practitioners, researchers, and policymakers on best practices, current challenges, and prospects. It aims to foster a transition towards more sustainable, efficient, and intelligent farming practices while encouraging the continued adoption and adaptation of new technologies.

## 1. Introduction

In today’s rapidly changing agricultural landscape, Precision Agriculture (PA) is emerging as a result of the strategic integration of smart sensors and advanced data analytics to guide agricultural decisions [1] in their application to factors such as fertilizers, water, pesticides, insecticides, seed, etc. The aim of PA is to optimize farming practices, increase resource efficiency, and minimize environmental impact [2]. At the same time, the current revolution in agriculture is propelled by a range of cutting-edge technologies, including remote sensing [3,4], GIS (Geographic Information Systems) [5,6,7], GPS (Global Positioning Systems) [8,9,10], big data analysis [11,12], the IoT [13,14], and AI [15,16,17]. These technological advances are playing a central role in the transformation of agricultural practices, offering solutions to optimize inputs, increase productivity, and mitigate environmental impact [18,19]. These advances include the development of IoT technology systems [20,21], the use of cloud computing, the use of wireless sensor networks, and the application of AI techniques such as machine learning [22,23]. All these advances contribute to the implementation of intelligent agricultural operations [24]. With the increasing adoption of these cutting-edge technologies in agriculture, the ability to characterize spatial variability and address the challenges that impede crop growth is becoming crucial to the success of PA [25,26].

**Figure 1:**
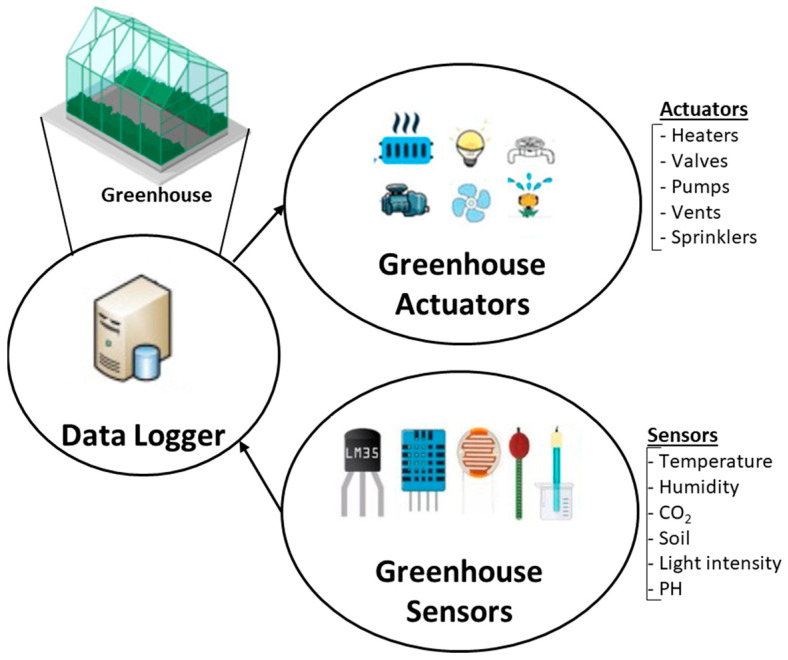
Smart greenhouse architecture diagram.

For the purposes of PA, predictive analytics software and forecasting systems harness agricultural data to offer farmers advice on soil management, crop rotation, and optimal planting and harvesting times [27]. Within this framework, sensor technology plays a crucial role in addressing various challenges related to PA [28]. Agricultural monitoring systems, powered by Wireless Sensor Networks (WSNs) deployed in cultivated areas, provide monitoring services to maintain optimal plant growth and enable early detection of conditions that could lead to widespread plant diseases [29,30,31]. In addition, smart irrigation systems, integrating IoT and sensor technology, are emerging as a solution to address the scarcity of clean water resources required by various plant crops, ensuring optimal use of water resources in PA [32,33]. Meeting the challenge of adapting platforms to the requirements of soilless cultivation in smart greenhouses, particularly in the context of moderate saline water use, is a key aspect of PA [34]. This challenge is effectively met by integrating IoT, cloud computing, and edge computing technologies. Figure 1 shows an architecture diagram for smart greenhouse applications in precision agriculture.

The literature review reveals an extensive range of techniques and tools used by researchers in PA using WSNs. Various platforms, such as Arduino, IMOTE2, MICAZ, MOSEK, the MDA300 sensor board, EZ430-RF2500, Indriya, MSP430, PIC16F877, ZKOS, MDA320, the CVX toolbox, MTS400, and the Veris mobile sensor platform, have been developed to integrate various sensors for the collection of crucial data information relating to PA parameters [35]. In particular, the Arduino platform has become a popular choice due to its affordability and ease of integration [36]. In addition, researchers have developed algorithms such as DCTA (Dynamic Converge cast Tree Algorithm) [37], OASNDFA (Optimized Algorithm of Sensor Node Deployment for intelligent Agricultural monitoring) [38], BOP (Beacon Only Period) [39], distributed localization algorithms [40], polynomial-time algorithms for node deployment [41], range-based localization algorithms [42], electromagnetic induction algorithms [43], MLR (Multiple Regression Analysis) [44], PCR (Principal Component Regression) [45], and PLSR (Partial Least Squares Regression) [46] methods for efficient monitoring and intelligent irrigation of crops. The review also highlights the introduction of new communication protocols, including the Fuzzy-based Energy Efficiency Protocol [47,48], APTEEN (Adaptive Periodic Threshold-sensitive Energy Efficient sensor Network protocol) [49,50], while operating systems such as Tiny OS and Tiny OS2.0 [51] are frequently adopted for sensor implementations in PA [35]. Table 1 shows an example of the platforms available in this context.

**Figure 2:**
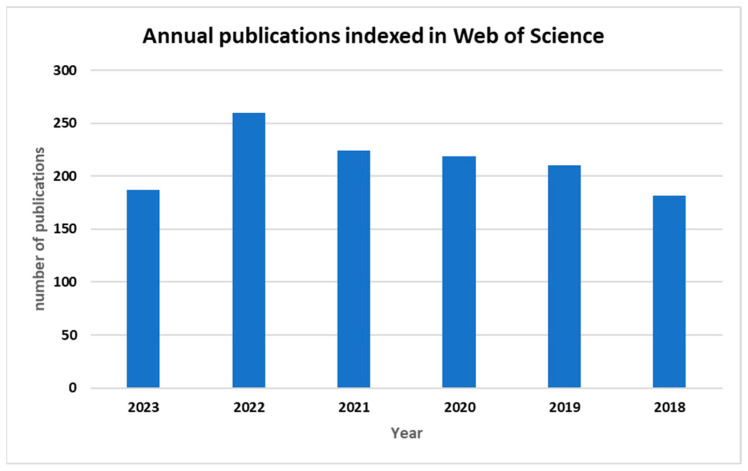
Annual publications indexed in Web of Science during the period 2018–2023.

In this context, this work presents a systematic literature review approach using the Web of Science database for the period 2018–2023. The search criteria are based on the TAK (Title, Abstract, and Keywords) approach [52], focusing on the keywords “sensor” and “precision agriculture”. Scientific articles were matched based on the specific context of greenhouses, excluding those related to general agriculture or other irrelevant applications for this review. The dataset comprises 1282 records distributed across publication years from 2018 to 2023. Notably, 2022 stands out with the highest record count at 20%, indicating a recent emphasis on publications from that year. The years 2021, 2020, and 2019 closely follow, with record percentages ranging from 16% to 17%, showcasing a consistent trend in recent years (Figure 2). Conversely, previous years exhibit diminishing record counts, with 2018 contributing the least at 14%. This analysis underscores a temporal evolution in the dataset, highlighting a concentration of records in recent years and a gradual decline in earlier publication years, as shown in the following figure. In this review, 99 articles, spanning various categories and concentrated in recent years, were selected.

**Figure 3:**
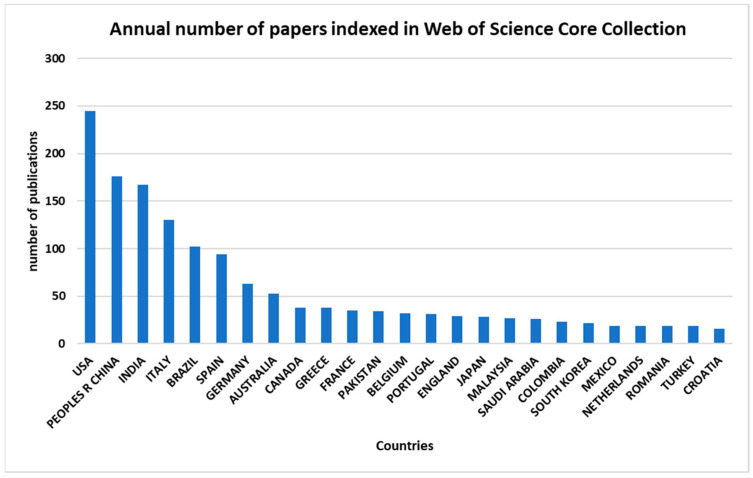
Annual distributions of papers indexed in Web of Science by country during the period 2018–2023.

The data also present a distribution of record counts across different countries or regions, with the United States (USA) having the highest record count, constituting 19% of the total, followed by the People’s Republic of China with 14%, and India with 13%. Italy, Brazil, and Spain also contribute significantly, with 10%, 8%, and 7%, respectively (Figure 3). The top countries show a diverse representation, indicating a global perspective in the dataset. The remaining countries each contribute varying percentages, with smaller contributions from several nations.

**Figure 4:**
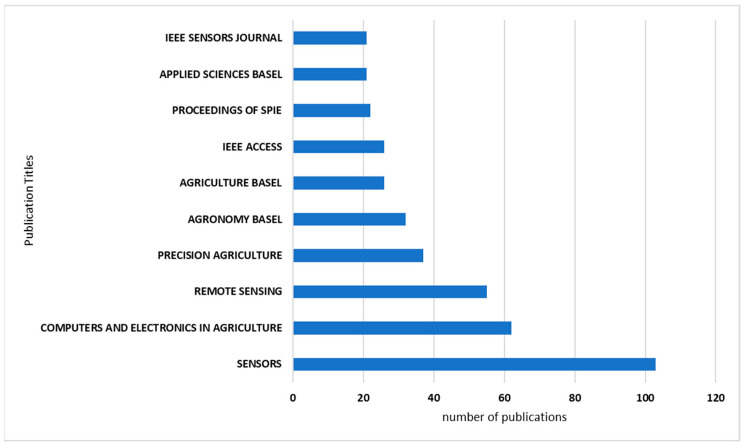
Annual publications of papers indexed in Web of Science by publication title during the period 2018–2023.

The data also illustrates the distribution of record counts across different publication titles, totaling 1282 records. The journal *Sensors* has the highest record count, accounting for 8% of the total, indicating a significant focus on sensor-related research. Following closely are *Computers and Electronics in Agriculture* with 5% and *Remote Sensing* with 4%. Other prominent titles include *Precision Agriculture*, *Agronomy-Basel*, and *Agriculture-Basel*, as shown in Figure 4.

**Figure 5:**
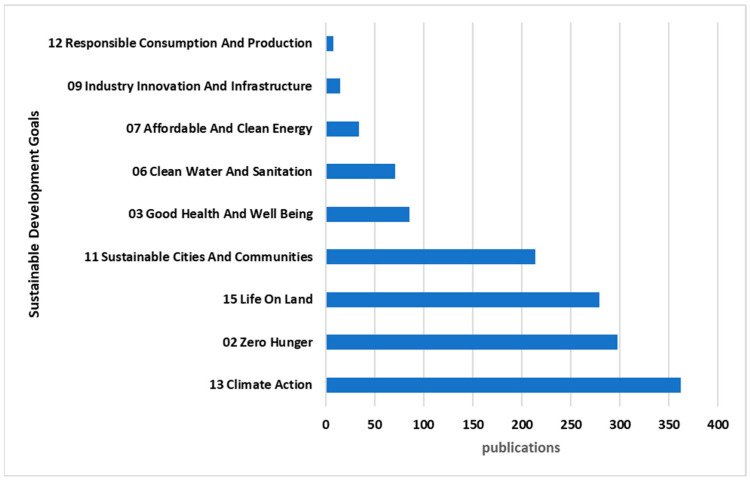
Annual publications of papers indexed in Web of Science by different Sustainable Development Goals during the period 2018–2023.

The data can also provide an overview of the distribution of records across different Sustainable Development Goals (SDGs), of which “13 Climate Action” stands out as the most prevalent goal, representing 28% of the total records, followed by “02 Zero Hunger” with 23%, and “15 Life on Land” with 22%. These high percentages indicate a significant focus on environmental and climate-related themes in the dataset. “11 Sustainable Cities and Communities” also has a notable representation at 17%. Other SDGs such as “03 Good Health and Well Being”, “06 Clean Water and Sanitation”, and “07 Affordable and Clean Energy” contribute to the dataset, albeit to a lesser extent. It is noteworthy that 20% of the records do not contain data on the analyzed SDG field (Figure 5).

This article aims to provide an overview of current smart sensors and smart data applications in PA, focusing on the monitoring and control of indoor microclimatic conditions in smart greenhouses. The article is structured as follows: Section 2 introduces various types of smart sensors crucial in PA. Subsequent sections delve into control and monitoring techniques specific to different applications. Section 3 details data collection methods and optimization techniques for monitoring and control in smart greenhouses. Section 4 explores the application of smart sensors in PA for indoor parameters such as soil moisture, temperature, air humidity, CO_2_, and light intensity. In addition, it provides case studies illustrating precision irrigation, fertilizer management, and pest and disease monitoring. Section 5 discusses the challenges and describes future directions for smart sensors in PA.

## 2. Types of Smart Sensors in Precision Agriculture

Today’s smart sensors are fundamental to data-driven decision-making in PA, facilitating farmers’ monitoring and optimization of various parameters vital to crop health and resource management. As mentioned in [53], agriculture relies on six key sensors, particularly in outdoor farming. These sensors include location and position sensors, essential for precise field operations; optical sensors, which measure soil properties at different light wavelengths; electrochemical sensors, providing information on soil pH and nutrient levels; mechanical sensors, offering information on soil compaction; dielectric/electromagnetic soil moisture sensors, determining soil moisture levels; and airflow sensors, measuring soil air permeability. In addition, the importance of Photosynthetically Active Radiation (PAR) sensors in optimizing plant growth conditions is discussed, as is the presentation of robotic sensors for crop weeding and spraying as effective and environmentally friendly alternatives to the traditional use of pesticides. This section gives an overview of the diverse range of intelligent sensors that have become indispensable tools in modern agriculture.

**Figure 6:**
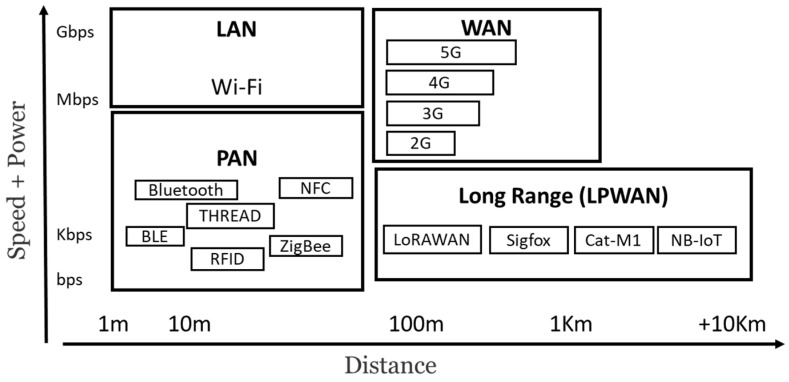
Range and rate comparisons among wireless communication technologies.

Generally, wireless communication technologies classified according to transmission distance can be divided into three categories: short-range (≤10 m), medium-range (10 m to 100 m), and long-range (≥100 m). Short-range technologies discussed include RFID, Bluetooth, and UWB (Ultra-Wide Band); each of these technologies is specifically designed for applications with limited proximity [54]. Wi-Fi and ZigBee are identified as major players in medium-range wireless communication technologies, offering reliable performance within a range of 10 to 100 m. The focus then shifts to long-range wireless communication technologies, in particular LPWA (Low-Power Wide-Area) options such as LoRa, NB-IoT, and Sigfox, which have emerged as alternatives to traditional cellular networks (2G/3G/4G/5G) [55]. LPWA technologies are characterized by low energy consumption, low data rates, wide coverage, and support for massive connections. Although LPWA technologies are not suitable for streaming audio or video data, they excel at connecting devices requiring minimal data transmission over long distances with extended battery life [56]. Figure 6 illustrates a comparison of range and speed among different wireless communication technologies.

In terms of comparative analysis, it highlights the trade-offs between power consumption, communication range, and data throughput for various wireless communication technologies. Notably, Bluetooth is identified as optimal for low power consumption, while ZigBee offers balanced range and data throughput [57]. LPWA technologies, such as LoRa and NB-IoT, are recommended for applications favoring long-range communications and low power consumption, even at the expense of lower data rates [58]. This comprehensive overview helps to understand the strengths and limitations of different wireless communication technologies, providing valuable information for a variety of applications. Table 2 shows a comparison of existing communication technologies along with their limitations.

a- Weather and Environmental Sensors

In the field, weather and environmental sensors provide real-time data on weather conditions, including temperature, humidity, wind speed, and precipitation. These sensors also measure environmental parameters such as atmospheric pressure and solar radiation. They are essential for forecasting and managing weather-related risks, optimizing irrigation, and ensuring the general well-being of crops.

The application of wireless sensor networks in greenhouses offers significant advances in crop management. Greenhouses provide an artificial environment for plants, effectively protecting them from weather, pests, and disease, reducing risk, and optimizing crop yields. The authors in [66] implement a wireless sensor network to monitor parameters such as temperature, humidity, and soil moisture, ensuring automatic control of climatic conditions. The data from each sensor node is transmitted to the cloud, where it can be monitored and analyzed. Actuators further enhance the system by adjusting growth parameters when anomalies are detected. This system provides 24/7 monitoring and control for consistent, optimized crop production, while users can access growth data in real time via a responsive web page set up on a local ESP32-based server, offering temperature and irrigation control based on predefined threshold values. The system architecture comprises a Wireless Sensor Network (WSN), a Network Coordinator (NC), and a Web Server (WS), ensuring modularity and scalability for efficient data collection and management. The integration of sensors such as the DHT11 temperature and humidity sensor, as well as resistive soil moisture sensors, enables accurate data acquisition for informed decision-making, improving greenhouse farming practices.

An automated smart greenhouse system [67], leveraging an Adaptive Neuro Fuzzy Inference System (ANFIS) and the IoT, presents an innovative solution for enhancing crop production. This system collects real-time data from sensors measuring four critical weather parameters: temperature, humidity, sunlight, and soil moisture. The gathered data serves as input for a fuzzy control system, which manipulates the information, and ANFIS predicts optimal weather parameter values. Farmers can monitor and adjust temperature and humidity through a mobile app. Data transmission occurs via GSM or TCP/IP, with IoT perception layer data being transferred to the application layer. Security measures are in place, including the detection of potential IoT perception layer attacks and their mitigation. The working procedures of various sensors in this automated greenhouse system are intricately designed to ensure precise environmental control. The DHT11 temperature and humidity sensor was used to measure ambient temperature and humidity every two hours during the day, and at four-hour intervals at night. These sensors connect to a high-performance 8-bit microcontroller, which then transmits the data to the FIS. If temperatures fall below 20 °C or rise above 30 °C, the microcontroller triggers a heater or cooling fan, maintaining the desired range. The humidity sensor, also using the DHT11 sensor, tracks humidity levels and activates exhaust fans if humidity exceeds 70% or fans if it falls below 65% RH. Soil moisture is managed by the YL-69 soil moisture sensor, prompting water pump activation when soil moisture drops below 70%. The Light-Dependent Resistor (LDR) sensor maintains optimal lighting conditions, switching lights on when insufficient light is detected. The system performs effectively, detecting attacks with a 93.62% accuracy rate, making it superior to existing systems. It is suitable for both large-scale and small-scale agriculture, offering precise soil moisture measurement and greenhouse maintenance. Customizable and efficient, the system combines IoT and fuzzy logic, enhancing learning efficiency and prediction accuracy. Future improvements may include nano-robotic sensors and predictive capabilities, ultimately making the system independent of human intervention.

The application of advanced sensor systems in modern agriculture, particularly in greenhouses, marks a significant technological advance that streamlines agricultural processes. This system [68] uses a wireless sensor network, including the DHT11 humidity and temperature sensor, and an intelligent LoWPAN gateway that connects the Zigbee network to the Internet, enabling continuous data transfer and analysis. The DHT11 sensor excels at measuring temperature and humidity with speed, accuracy, and high resolution, making it a valuable asset in greenhouse environments. The data collected by these sensors is transmitted via the ZigBee network to an intelligent gateway and then sent via mobile data communication to a web service. Intelligent software applications analyze this data, enabling automated responses via actuators to create an optimized microclimate within the greenhouse. This innovative system improves the efficiency of greenhouse farming while promoting higher-quality production at a time when technology is revolutionizing agriculture.

The deployment of a ONENET-based remote monitoring and control system for greenhouse environments represents a significant step forward in realizing the potential of the IoT in agriculture. The system presented by [69] features the efficient integration of advanced sensors using the STM32 MCU to monitor air temperature, humidity, light intensity, soil temperature, and moisture in the greenhouse. It integrates a relay control unit and a ONENET IoT cloud platform, enabling remote greenhouse management via computer or mobile devices. In addition to the DHT22 temperature and humidity sensor, the MG811 CO_2_ concentration sensor, the BH1750 lighting sensor, and the SHT-20X soil temperature and humidity sensor, the information acquisition unit includes the STM32 central controller overseeing data collection and relay control. Data collected by the sensors is processed and relayed to the ONENET cloud platform via the ESP8266 wireless WIFI module, which then connects to the router. This seamless integration enables real-time monitoring and control of greenhouse environmental data, accessible via an intuitive dashboard on the ONENET cloud services platform, optimizing the management of agricultural conditions. Practical results highlight the system’s advantages, including high detection accuracy, simple design, and cost-effectiveness. Leveraging cloud-based data storage and real-time monitoring, the system harnesses IoT technology to streamline environmental control in greenhouses, marking a substantial step towards optimizing agricultural practices.

This research by [70] highlights the disadvantages of current IoT-based agricultural applications, particularly in terms of their environmental and health implications. To address these concerns, the study introduces Green IoT (G-IoT) and green nanotechnology, aiming to develop a cost-effective, real-time precision agricultural monitoring system. This system aims to minimize energy consumption, reduce Greenhouse Gas (GHG) emissions, and provide a user-friendly interface, enabling farmers to monitor various agricultural parameters such as weather, water, soil, pest detection, intrusion detection, and fire detection remotely via smartphones. Unlike many existing PA monitoring systems that focus solely on irrigation, the proposed system incorporates LoRa technology for long-range communication, reducing energy consumption compared with ZigBee, Wi-Fi, and GSM protocols. The study highlights the importance of periodically monitoring various parameters to inform farmers and avoid wasting resources, thus acting as a decision support system. The integration of G-IoT tools and LoRa technology in the proposed system contributes to its cost-effectiveness, energy efficiency, and accessibility for farmers with no specialist training.

b- Soil Sensors (moisture, temperature, nutrient levels)

Soil sensors come in a variety of forms and measure essential soil properties such as moisture content, nutrient levels (nitrogen, potassium, and phosphorus), temperature, and PH. These sensors constantly monitor soil conditions to guide precise irrigation and fertilization practices, avoiding overuse of resources and ensuring optimum nutrient availability for crops [71].

The integration of embedded systems in agriculture, particularly in the context of smart greenhouse farming, is a vital and transformative development in the agricultural sector. This innovative solution aims to combat climate-related challenges and optimize crop yields. The smart greenhouse system, detailed in [72], offers localized automated agricultural control, enabling precise regulation of temperature, soil moisture, and light intensity to create an ideal environment for crop growth. Furthermore, the system allows remote greenhouse monitoring via the Internet, providing real-time data on crucial parameters. The system takes advantage of long-range connectivity, uses sensors for soil moisture, light, and temperature, and carries out automation by interfacing with actuators via a Nucleo Mbed STM board. Collected data is processed and uploaded to the cloud, facilitating remote monitoring for farmers. The integration of Microsoft Azure Cloud and Power BI ensures efficient data visualization and analysis, giving farmers the tools they need for informed decision-making and effective greenhouse management. This article presents the potential of integrated systems to revolutionize agriculture for improved productivity and sustainability.

In [73], an IoT-enabled Wireless Sensor Network (WSN) framework was utilized, incorporating various soil sensors, environmental sensors, CO_2_ sensors, and light intensity sensors to gather real-time agricultural data. These IoT-enabled WSN nodes collected and stored data, and a neural network-based prediction model was employed to optimize irrigation by managing water valves based on soil water requirement forecasts. The study conducted a comparative analysis of optimization techniques and found that the Variable Learning Rate Gradient Descent (VLRGD) outperformed Gradient Descent (GD) in soil moisture prediction. The research also generated a comprehensive soil moisture requirement map using interpolation and a Structural Similarity Index (SSIM)-based approach, leading to successful irrigation valve control commands based on fuzzy logic weather modeling to meet uniform irrigation needs under various weather conditions. The experiment was conducted in Bhubaneswar, India, on a farm with specific soil characteristics, and the results indicate the effectiveness of the proposed system for PA. Soil quality was assessed through laboratory analysis of a 3.5 kg soil sample, revealing dry bulk density and consistency values of about 1.43 g/cm^−3^ and 0.46 g/cm^−3^, respectively. The study deployed a WSN using multi-hop mesh network topology to interconnect all nodes. Each WSN node included an eight-channel Analog-to-Digital Converter (ADC) interfacing with various sensors, and communication occurred via an RS232 interface between the microcontroller (ATMEGA328P-PU) and Zigbee S2 units configured in a mesh network. The irrigation system employed sprinkler pipelines spaced at 0.41 m, with sprinkler heads delivering a flow rate of 2.2 L per hour (Lh^−1^).

In [74], the authors assess the potential of portable Laser-Induced Breakdown Spectroscopy (LIBS) for determining total mass fractions of major nutrients (Ca, K, Mg, N, P) and trace elements (Mn, Fe) in soil, as well as other parameters such as humus content, soil pH, and plant-available P content. To address the dependence of LIBS nutrient quantification on the soil matrix, various multivariate regression methods, including PLSR, least absolute shrinkage and selection operator regression (Lasso), and Gaussian Process Regression (GPR), were used for calibration and prediction. Optimal results were obtained for Ca, K, Mg, and Fe, while lower Mn concentrations and limited lines with low intensities affected the accuracy of N and P predictions. The study also explores the prediction of non-element soil parameters such as pH, with Lasso and GPR outperforming PLSR, and examines various methods of data pre-processing.

Another study [75] introduces AgriTalk, an affordable IoT platform designed for precise soil cultivation in agriculture. Turmeric cultivation experiments conducted with AgriTalk demonstrate a significant improvement in turmeric quality, particularly achieving curcumin concentrations ranging from 4500 to 5500 mg/100 g—five times higher than existing products. The paper illustrates the intuitive configuration of connections between sensors and actuators to achieve desired farming intelligence and the effective maintenance of AgriTalk for precision farming applications. Through measurement, analytic analysis, and simulation experiments, the research delves into the IoT message delays of AgriTalk. In particular, the findings reveal that the delays for automatic control and automatic-manual control switching, even over long distances (more than 30 km), are very brief (less than 0.2 s), demonstrating AgriTalk’s ability to promptly respond to quick and dynamic changes in the field environment during soil cultivation.

In another context, the authors of [76] address the need for predicting path loss in Wireless Underground Sensor Networks (WUSN) to optimize network performance. The proposed model, WUSN-PLM, relies on an accurate forecast of the Complex Dielectric Constant (CDC) to assess path loss in various communication scenarios (Underground-to-Underground, Underground Aboveground, and Aboveground Underground). WUSN-PLM considers reflective and refractive wave attenuation based on the burial depth of sensor nodes. Evaluation of WUSN-PLM involves extensive real-world measurements using different transceiver pairs in the botanic garden of the University Cheikh Anta Diop in Senegal. The results demonstrate that WUSN-PLM surpasses existing path loss models across various communication types, providing a precise and balanced accuracy of 87.13% and 85%, respectively, for deployment on cost-effective sensors.

c- Crop Health Sensors (NDVI cameras, chlorophyll meters)

The use of crop health sensors facilitates effective early detection of stress factors, nutrient deficiencies, and diseases, enabling rapid intervention and improved yields. These sensors assess crop vitality through the analysis of indicators such as leaf chlorophyll content, vegetation indices, and canopy temperature, most often using techniques such as the Normalized Difference Vegetation Index (NDVI) [77,78].

The authors of [79] focus on the application of Unmanned Aerial Vehicles (UAVs) and remote sensing techniques in South African PA to improve crop monitoring for smallholder farmers. The research uses multi-spectral UAV imagery and a random forest machine-learning algorithm to assess the chlorophyll content of maize at different growth stages. Using near-infrared and red-edge wavelength bands, as well as derived vegetation indices, the study successfully estimates chlorophyll content, providing crucial information on the health of maize during phenological stages. The random forest model was performed optimally, particularly during the early reproductive, late vegetative, and early vegetative growth stages. The results highlight the spatial heterogeneity of chlorophyll within the maize field, providing valuable information for decision-making and farm management. This research establishes the importance of integrating drone imagery and machine learning algorithms in PA to help small-scale farmers maintain crop health and productivity.

In the same context, a study [80] explores the integration of NDVI in automated greenhouses to improve PA. While automated greenhouses traditionally focus on environmental and soil variables, direct vegetation data, crucial for assessing crop health, is often neglected. The research aims to evaluate the integration of two types of NDVI measurement instruments into a greenhouse ecosystem, focusing on the correlation between NDVI levels and plant health. The correlation coefficients for healthy and stressed plants indicate the sensors’ reliability in measuring NDVI, with minor deviations noted. Despite slight offsets observed for lower NDVI measurements, the correlation remained consistent and even slightly improved for stressed plants, suggesting that these variations do not significantly affect the sensors’ reliability. The study highlights the importance of taking into account operational differences between sensors, such as data transmission rates, and highlights the pedagogical value of interdisciplinary research in the context of automated greenhouse technology.

The authors of [81] address the limited application of remote sensing methods to assess crop vigor and yields in sub-Saharan Africa, mainly due to the constraints associated with satellite imagery. Using UAVs as a viable alternative, the study presents the effectiveness of a vegetation index derived from UAV imagery for assessing maize crop vigor and yields at different growth stages. The study, based on a quadcopter equipped with consumer cameras, including one modified for near-infrared capture, reveals that drone-derived GNDVI (Green Normalized Difference Vegetation Index) serves as a superior indicator of crop vigor and a more accurate estimator of yields compared to traditional field methods such as SPAD readings. The results highlight the potential of drone-based remote sensing, in particular, GNDVI, as a reliable and timely predictor of crop performance and yield, even in the context of small, complex farms in SSA.

The authors of [82] present a real-time computer vision system designed for variable-rate agrochemical spraying to overcome the challenges associated with traditional spraying methods, such as overdosing or underdosing. The system uses a Random Forest classifier for weed/crop detection and classification. Training of the classification model with a customized data set precedes its deployment in the field of practical testing. The agrochemical spraying process uses a Pulse-Width Modulation (PWM)-based fluid flow control system, ensuring precise application of the required agrochemical quantities guided by the vision-based feedback system. The results of several field tests confirm the effectiveness of the proposed vision-based agrochemical spraying framework in achieving real-time precision.

d- GPS and Remote Sensing Technologies

The use of GPS and remote sensing technologies such as satellites and drones provides access to detailed, high-resolution spatial information. These advanced tools facilitate accurate mapping, crop yield monitoring, and the creation of precisely defined field boundaries [83]. They represent invaluable tools for assessing crop performance and improving the efficient allocation of resources in agriculture [84]. In this context, numerous studies have reviewed remote sensing techniques and their applications in PA. Several studies have concentrated on specific application areas such as soil and crop management, Evapotranspiration (ET) management, and disease and pest management, while others have covered more than one application area [85,86,87,88]. Table 3 provides examples of satellites used for PA and their applications.

In the PA field, drones have gained importance, being used for remote sensing tasks such as photo and video capture, as well as functions such as fertilization or animal deterrence. The authors of [97] assess the viability of using remote-sensing drones as mobile gateways, taking advantage of their pre-set flight plans. In contrast to existing proposals, this study innovatively assesses the performance of UAVs as low-cost remote sensing tools and gateway nodes, collecting data from field-deployed nodes in PA systems for crop monitoring. Simulations in various scenarios, taking into account factors such as drone speed, flight height, node density, and antenna coverage, were carried out to verify the feasibility of accurate data transmission. The results indicate specific combinations, such as lower speed, 24 m flight height, and 25 m antenna coverage, for optimal connectivity under maximum node density, while various combinations offer good and optimal connectivity for other node densities. This research provides valuable information for selecting optimal drone parameters for efficient Wi-Fi data transmission in PA systems.

In [98], the challenges of deploying PA infrastructure, in particular Decision Support Systems (DSS), in small-farm contexts due to costs and technical limitations are discussed. It exploits freely available satellite data (Sentinel-2A) and the SNAP toolbox in the context of Open-Source Remote Sensing (OSRS) to demonstrate the potential for monitoring crop health and development. Using the Tono irrigation system in Ghana as a case study, this research demonstrates the effectiveness of OSRS in providing valuable information for agricultural management and resource allocation decision-making. SNAP Toolbox’s vegetation index algorithms are proving capable of accurately identifying crucial crop conditions such as chlorophyll content, nitrogen status, pest and disease infestations, and water requirements. However, for this innovative OSRS-based monitoring system to be cost-effective, the article highlights the need for basic training for scheme managers and extension workers in interpreting the results of OSRS analysis. Implementing OSRS as a DSS for smallholder farmers in resource-poor contexts has significant potential to reduce costs, optimize resource allocation, and improve yields.

The authors of [99] investigate the estimation of durum wheat yield using various technologies and data processing methods. The use of an advanced data cleaning technique on yield monitoring system data results in close agreement between yield monitoring and manually sampled data. The study assesses the potential of Sentinel-2 and Landsat-8 imagery in PA to address production variability within the field, and determines the optimal timing for remote sensing to correlate with durum wheat yield. In the study, NDVI (Normalized Difference Vegetation Index) was compared with yield monitoring data, revealing significant and highly positive linear relationships (ranging from 0.54 to 0.74), explaining most of the within-field variability. Application of remote sensing data with these methods can effectively assess durum wheat yield, describe spatial variability, provide location-specific management, enhance productivity, save time, and offer a potential alternative solution for traditional farming practices.

The authors of [100] discuss the challenges associated with Ground Control Points (GCPs) in agricultural remote sensing using UAVs. Conventional stationary GCPs are labor-intensive and time-consuming in large terrain configurations. The authors present an autonomous mobile GCP and a collaborative strategy to improve the efficiency and accuracy of UAV-based data collection. This system’s ability to automatically follow the trajectory was demonstrated in preliminary tests, reducing the RMSE (Root Mean Square Error) lateral deviation from 34.3 cm to 15.6 cm. These tests also validated the feasibility of moving reflectance reference panels without affecting pixel values in mosaic images. In field tests, the autonomous mobile GCP successfully collaborated with the UAV in real time, outperforming conventional calibration methods employing stationary GCPs in terms of georeferencing, radiometric calibration, height calibration, and temperature calibration.

The authors of [101] address the challenge of accurate georeferencing of orthomosaics obtained from UAV images, a critical factor for informed decision-making and the implementation of precise actions in crops. The study also assesses the performance of Real-Time Kinematics (RTK) and Virtual RTK during UAV flights, intending to produce high precision orthomosaics applicable to precision farming processes. The results demonstrate a significant reduction in positioning errors, up to 98%, compared with direct GPS georeferencing. The accuracy assessment involves a static survey using the Mallegan Professional DGPS ProMark3 for precise location measurements of seven control points, which are then compared with estimated orthomosaic coordinates.

e- Water Quality Sensors

Water quality sensors are responsible for monitoring various aspects of irrigation water, including salinity, dissolved oxygen levels, and turbidity. These sensors play an essential role in preserving the suitability of irrigation water for cultivation and protecting against soil deterioration caused by excess salts or contaminants [102].

The application of sensors in PA is a pivotal component of automated irrigation systems, addressing the challenges of overwatering and underwatering crops, thereby optimizing resource utilization. This system [103] leverages wireless technology and a Wireless Sensor Network (WSN) with IoT principles, ensuring efficient communication between sensor nodes and the central control unit. Temperature, humidity, soil moisture, and water tank levels are continuously monitored, and the data is transmitted to users via GSM communication. This smart irrigation system caters to the specific water requirements of different crops in various areas, facilitating precise and cost-effective agriculture management, particularly for Indian farmers. The WSN, coupled with IoT, enables remote data control and analysis through a user-friendly Android application, offering both automated and manual operation options. The system architecture, primarily centered around the nodeMCU, connects sensors and hardware components while integrating GSM and Wi-Fi modules for seamless communication and data storage, ultimately enhancing the efficiency of PA practices.

Efficient water use in agriculture is a crucial challenge addressed by modern technologies, in particular through Irrigation Advisory Services (IAS) and Decision DSS. The authors of [104] present the results of the project “An Advanced Low-cost System for agricultural Irrigation Support—LCIS”. The LCIS-DSS, developed as part of this project, integrates three methodologies: W-Tens (in situ soil sensor), IRRISAT^®^ (remote sensing), and W-Mod (Water balance simulation Modeling). The evaluation carried out on maize crops in southern Italy during the 2018 season assesses the predictive performance of the tools as well as their advantages and disadvantages, taking into account applicability on a spatial scale as well as costs and complexity of use. While all three approaches contribute to maximizing corn production, the in-situ soil sensor method (W-Tens) delivers 40% more water, underscoring its efficiency, while IRRISAT^®^ and W-Mod excel in Irrigation Water Use Efficiency (IWUE). IRRISAT^®^ stands out in particular for its ability to operate without spatial soil information. The study highlights the potential of integrated tools to improve agricultural water use efficiency, recognizing the role of field sensors and remote sensing in this context.

The authors of [105] propose a scalable Wireless Sensor Network (WSN) architecture integrated with the IoT to Enhance Precision Agriculture and Farming (PAF) practices, particularly focusing on efficient irrigation water resource management. The system relies on IoT for communication and data processing in remote agricultural areas. Throughput maximization, latency minimization, high Signal-To-Noise Ratio (SNR), minimum mean square error, and improved coverage area are evaluated to optimize the WSN structure. Results demonstrate the superiority of this approach over conventional IoT-based PAF systems. Furthermore, the paper introduces a Smart Irrigation System for PA and Farming (SIS-PAF) utilizing IoT and cloud computing to address water scarcity issues by reducing irrigation wastage. The system incorporates hardware components like Arduino Uno, moisture sensors, and a GSM modem for wireless communication. Data collected from Arduino is transmitted via GSM to a webpage, allowing farmers in remote areas to monitor and control irrigation, adjusting it based on soil moisture levels. Machine Learning (ML) is employed in a two-phase process to optimize water supply, and ultrasonic sensors track plant growth. Overall, this automated system empowers farmers with real-time data on soil conditions, crop growth, and environmental parameters, ultimately improving crop productivity and water resource utilization in PA.

A proposed study in [106] presents a low-power wireless sensor network using the LoRaWAN protocol designed for cost-effective PA applications, with a focus on greenhouse detection and actuation. The research focuses on a modular system built from readily available components and open-source software libraries. The experiment validates the system’s reliability through continuous testing with two natural soils, Silty Loam and Loamy Sand. In particular, the study compares the performance of a low-cost Volumetric Water Content (VWC) sensor with a reference sensor from Sentek, revealing a non-constant sensitivity for the former. The proposed system uses a new procedure to optimize parameters and extract reliable VWC values, introducing a unique approach to address the sensor’s lack of linearity. The layered architecture integrates wireless nodes, a LoRaWAN network, and cloud-based applications, offering real-time data visualization via a graphical user interface. While acknowledging the need for further optimization, the study presents a promising method for improving the performance of low-cost sensors in PA applications.

The authors in [107] present a new approach, the Deep Learning-based IoT intelligent irrigation system for precision agriculture (DLiSA). Using a Long Short-Term Memory Network (LSTM), DLiSA predicts volumetric soil moisture content, irrigation timing, and spatial water distribution for arable land one day in advance. The integrated feedback system is designed to maintain functionality in a variety of weather conditions and regions. Simulation results demonstrate that DLiSA outperforms state-of-the-art models in terms of judicious water use in experimental agricultural areas. The focus is on crucial agricultural aspects such as water conservation and control of irrigation periods. Calibration and testing throughout one and a half years at three sites demonstrate DLiSA’s reliability, and its performance compares favorably with existing models for predicting soil moisture content over one month. The authors highlight the significant water savings achieved by the proposed model compared with alternative models, keeping the soil moisture deficit within acceptable limits. Future research aims to improve the predictive capabilities of the irrigation scheduler by integrating the prediction of rainfall depth, further optimizing water conservation through efficient use of rainfall.

f- Pest and Disease Detection Sensors

Some sophisticated sensors have the ability to identify the existence of pests and diseases in crops. They use methods such as image recognition, spectroscopy, or other techniques to discern the presence of pathogens or pests, enabling rapid action to mitigate potential damage [108,109].

The study conducted in [110] delves deeper into the field of intelligent agriculture by introducing an embedded system enriched with ML functionalities for the continuous detection of pest infestations in fruit orchards. Although artificial intelligence has made significant inroads in various sectors, including agriculture, the focus on low-power detection devices with embedded ML capabilities remains rare in smart agricultural applications. The paper focuses on the critical problem of biotic stress affecting crop yields and addresses it through the development of a smart trap using ML algorithms for rapid pest detection in apple orchards. The integrated solution incorporates a Neural Accelerator, enabling image capture and processing in pheromone-based traps. The system has trained and deployed three ML algorithms, demonstrating the platform’s versatility. The proposed approach ensures extended battery life thanks to energy recovery components, enabling continuous, autonomous detection of pest infestations without the need for frequent farmer intervention. The hardware architecture, implemented on a Raspberry Pi microcomputer, includes a camera as an image sensor and an Intel Neural Compute Stick (NCS) for optimized inference execution and reduced power consumption. The study makes a significant contribution to the field of PA by introducing an efficient, autonomous system for detecting pests in orchards.

A study in [111] highlights the growing need for PA to meet global food demand while minimizing environmental impact and optimizing resource use. Focusing on the challenge of cost-effective technology integration for farmers, the paper presents a wireless sensor network using low-cost soil moisture sensors to improve irrigation processes in PA. Each wireless node comprises four soil moisture sensors measuring moisture at different depths using a mechanism based on mutual induction. Several prototypes were tested, with the optimum design featuring a 1:2 winding ratio with 15 and 30 arrows at 93 kHz. A specific communication protocol was developed to enhance system performance. The proposed system was tested on a real cultivated plot, evaluating coverage and the Received Signal Strength Indicator (RSSI) to assess losses due to vegetation. The paper also details the network protocol, message exchange, data collection, and control algorithm, focusing on a distributed network structure with sensor and actuator nodes. This comprehensive approach addresses the need for affordable and reliable precision farming systems, marking a significant contribution in this field.

In the same context, the authors of [112] address a prevalent problem in apple orchards, focusing on combating the threat of the codling moth, an important pest for apple crops. Leveraging advances in IoT sensing devices with embedded ML algorithms, the proposed system offers extensive real-time data collection and analysis capabilities, specifically targeting codling moth detection. The innovative approach uses neural network algorithms close to sensors that automatically identify codling moths. The system captures trap images, pre-processes them, and uses a neural network to classify the insects, rapidly informing the farmer as soon as they are detected. In addition, the application runs on a low-power platform, using a compact solar panel for energy autonomy, guaranteeing continuous, unattended operation over low-power wide-area networks. The study highlights the importance of a low-power platform for rapid IoT prototyping with ML algorithms. In the study, a Raspberry Pi3 board and an Intel Movidius NCS are used for pre-processing and implementation of the neural network, respectively. A detailed analysis of the network model, parameters, and limitations in light of hardware constraints is provided. System performance is thoroughly evaluated, with discussions on power consumption aimed at achieving a zero-energy balance.

## 3. Data Collection and Management Techniques

To create effective monitoring solutions, there are a number of potential benefits to be gained from integrating agronomic knowledge with emerging technologies such as the IoT, cloud computing, and smart systems. There are some commonalities between various studies, including the use of low-cost devices, low-power sensor nodes (Arduino^®^ and ESP8266 microcontrollers), and communication protocols such as ZigBee and IEEE 802.11. Gateways play a crucial role in data aggregation and local processing within the Fog computing concept, with some studies transmitting data directly to remote web/cloud services. In remote locations, GSM/GPRS is a widespread solution. On the server side, data analysis services are increasingly accessible to end-users via mobile or PC applications. The high processing capacity of the cloud enables efficient data handling, particularly when processing large volumes from numerous low-cost devices. In particular, there is a growing trend towards proximity surveillance scenarios involving the use of images, introducing increased processing requirements for capture elements and transmission via remote servers [113].

Collecting data in the field is essential for efficient precision farming, ensuring the optimal use of resources such as water, fertilizers, and pesticides. The integration of GPS receivers on farm equipment facilitates precise positioning in global coordinates, a crucial aspect for site-specific management and instrument navigation. The use of multiple positioning sensors, including GPS and inertial sensors, improves the accuracy of vehicle navigation in the agricultural sector. The adoption of intelligent farming techniques involves automated data collection via computer-aided systems, in which wireless technology plays a crucial role in eliminating cables, thus reducing maintenance problems and costs. Data management tools hosted on cloud servers enable fast and accurate operations, with analysis tools and pre-configured algorithms improving overall efficiency. The authors of [53] introduce the concept of TV Whitespace (TVWS), unused bandwidth between active TV channels, as a solution for wireless data transmission in agriculture. TVWS, often overlooked, offers the possibility of efficient data transmission, with applications in remote sensor data collection, farm management, and drone imagery. The use of TVWS in the development of 5G transmission further underlines its potential to revolutionize PA by offering faster transmission speeds and extended range compared with traditional communication channels.

a. Data Acquisition Systems

To examine the challenges and potential of UAV-based multispectral remote sensing in PA, researchers in [114] used a narrowband Mini-MCA6 camera and a broadband Sequoia camera mounted on a micro-UAV for data collection in corn fields. The research highlights the importance of appropriate calibration methods, in particular the non-linear sub-band empirical line method, for accurate reflectance values, with the Mini-MCA6 camera proving more accurate in the visible bands. However, the accuracy of the Vegetation Indices (VI) does not depend entirely on reflectance accuracy, highlighting the variations between cameras. Mini-MCA6’s NDVI outperforms Sequoia’s, while Sequoia excels in red-edge NDVI (reNDVI). At the plot level, reNDVI outperformed NDVI in predicting Soil Plant Analysis Development (SPAD) values under different nitrogen treatments. The study highlights the instructive role of multispectral UAV remote sensing in PA but calls for further progress in calibration methods, post-processing techniques, and robust quantitative surveys.

The authors of [115] address the importance of grape quality and the drying process in raisin production, focusing particularly on southern Azerbaijan, a crucial region for grape cultivation. Recognizing the predominance of traditional vineyards and obsolete drying structures, the research highlights the need for modernization to improve quality and productivity without imposing excessive costs on traditional agriculture. To achieve this, the study proposes the implementation of a Wireless Sensor Network (WSN) system for real-time remote monitoring of micrometeorological parameters in three geographically dispersed vineyards. The WSN system facilitates data collection, enabling farmers to make informed decisions about irrigation scheduling, disease prevention, and optimal harvest times. The system integrates agrometeorological sensors in vineyards and drying structures with a gateway for data collection and wireless transmission to a central server. In addition, an online warning system alerts farmers in real time by SMS. Field experience validates the system’s functionality, demonstrating its effectiveness in the development of information systems for precision viticulture.

The authors of [116] explore the integration of computing technologies, including the IoT, WSNs, data analytics, and machine learning, in the context of agriculture, focusing on the prediction of apple diseases in orchards in the Kashmir Valley. The study proposes a predictive model using data analytics and ML within an IoT system. In addition, a local survey assesses farmers’ views on emerging technologies for PA. The practical implementation involves setting up a WSN with sensors and a gateway to capture data in apple orchards. Six sensors, each comprising an IRIS with an MTS 420 sensor board, cover specific areas of the orchard. The gateway, using MIB 520 with an IRIS, facilitates connectivity and communication. The document details the programming steps using the MoteConfig application for IRIS and MIB 520, ensuring correct configuration for efficient data collection and analysis.

b. Wireless Sensor Networks

The evolution of modern wireless communications and information technologies provides the foundation for PA. The authors of [117] systematically explore viable wireless communication technologies for PA through an analysis of agricultural application scenarios and experimental tests. The study presents the potential of three Wireless Sensor Network (WSN) architectures based on NB-IoT (narrowband), LoRa, and ZigBee wireless communication technologies for precision farming applications. Their feasibility is rigorously tested, and comparative evaluations are carried out by measuring the normal communication time and power consumption levels achieved by these wireless communication technologies. A comprehensive field test and analysis reveals that ZigBee is the optimal choice for agricultural plant monitoring, while LoRa and NB-IoT emerge as two wireless communication technologies suitable for agricultural field scenarios. The research provides valuable information on the selection of wireless technologies suited to the specific needs of PA.

Artificial Intelligence has made significant progress in various monitoring and control applications, particularly in the agricultural field. However, the development of low-power sensing devices incorporating AI remains fragmented in current research. The authors of [118] present an embedded system that integrates AI for continuous analysis and on-site prediction of plant leaf growth dynamics. The system uses an integrated low-power sensing system combined with a Graphics Processing Unit (GPU) capable of running neural network-based AI applications on board. A Recurrent Neural Network (RNN), in particular the Long-Term Memory Network (LSTM), is the main component of this system. In addition, the proposed approach ensures autonomous operation for up to 180 days on a standard Li-ion battery, leveraging state-of-the-art mobile graphics chips for intelligent analysis and monitoring of autonomous devices. For the experiment, the authors carried out meticulous calibration procedures for both the camera and the algorithm used to calculate leaf area, thus ensuring the accuracy of leaf area determination and minimizing errors in different regions of the camera’s field of view.

With the aim of improving resource utilization by providing real-time data for informed decision-making, a study [119] presents a new approach to agricultural and irrigation monitoring via a Wireless Sensor and Actuator Network (WSAN). The Agrinex wireless network uses a mesh configuration of field nodes serving as sensors for soil moisture, temperature, and humidity, and actuators regulating drip irrigation valves. The dynamic mesh network enables sensor nodes to self-organize in response to changes in the network. The irrigation actuator integrates an electronically controlled actuator and valve, guaranteeing energy efficiency thanks to the use of a rectifier (IRF530). The system presents a promising framework for WSAN applications, particularly in agriculture, highlighting the integration of components for efficient data processing and actuation in the Agrinex system.

The authors of [120] explore the challenges and solutions involved in implementing PA in greenhouses, focusing on the deployment of a LoRaWAN-based sensor network. Conducting real-time experiments at a research center in Belgium, the study presents a dashboard for data visualization, evaluates energy consumption in LoRaWAN communication, and tests different types of boxes. One notable discovery is the significant impact of temperature and humidity on sensor readings, addressed through an innovative airflow housing design. Focusing on energy-saving IoT strategies, the paper discusses implications for sensor enclosure design, advocates airflow enclosures, and proposes sequential steps for WSN deployment and monitoring. The in-depth study demonstrates the deployment of a LoRaWAN network to monitor tomato crops, highlighting the importance of continuous environmental data monitoring in PA. Future directions include efforts to improve the energy efficiency of the LoRaWAN network through energy recovery methods.

c. Cloud-Based Data Storage and Management

The researchers in [121] present an innovative IoT-based agricultural monitoring system with a cloud-based mobile application designed to enable farmers to easily observe and analyze their farms in real time. The system integrates various agricultural sensors such as humidity, temperature, and moisture with the Raspberry Pi, storing the data on the ThingSpeak cloud for future forecasting and improving crop quality. In addition, a Passive Infrared Sensor (PIR) is integrated to monitor potential intrusions. The study focuses on live monitoring of sensor values via cloud computing and a mobile application, providing a cost-effective, user-friendly solution that enables farmers to manage their farms efficiently and improve crop quality by minimizing wasted energy and resources. The proposed system, successfully implemented and tested, combines Raspberry Pi technology, IoT, and a cloud-based software architecture to streamline farming tasks and information processing. The ThingView mobile app serves as a graphical user interface compatible with Android and iOS operating systems, offering a complete solution for data visualization and farm monitoring. Interconnected components, including various sensors and a microcontroller board, facilitate the seamless flow, processing, and storage of data in the cloud. The connections between sensors and processors highlight the integration of sensors such as DHT11, PIR, and soil moisture sensors with the Raspberry Pi, demonstrating the practical implementation of the proposed system.

The study presented in [122] addresses the challenges of traditional detection technologies for pest and disease detection in specialty crops, proposing the use of small UAVs equipped with a variety of sensors. The cloud and AI application developed, Agroview, facilitates fast and accurate processing, analysis, and visualization of data collected from UAVs. In addition, it offers functionalities such as plant and gap detection, measurement of plant height and canopy size, and the creation of plant health maps. The application is applied to assess the phenotypic characteristics of citrus fruits, demonstrating its effectiveness with a low percentage Mean Absolute Percentage Error (MAPE) in detecting trees and estimating tree height and canopy size. The study focuses on the usefulness of cloud-based solutions, specifically using Amazon Web Services (AWS), to support the scalable and efficient deployment of Agroview, with different AWS instances allocated to specific processing tasks. The architecture includes a main application control machine, an assembly engine for CPU-intensive tasks, and a GPU-intensive instance for the tree detection algorithm. Data storage on AWS enables scalability and memory upgrades as required. Agroview offers a consistent, cost-effective, and fast solution for field surveys and plant phenotyping in specialty crop production.

In response to the urgent need to improve agricultural practices in India, the study reported in [123] takes advantage of the Internet of Everything (IoE) to gather crucial data on the agricultural habitat, such as soil moisture, humidity, light, and temperature. The IoE system uses sensor nodes capable of transmitting monitoring data to the cloud. Then, data mining principles are applied to reveal meaningful patterns within the environmental data collected by the sensor network. The research, carried out as a use case on a farm in Chennai, introduces the peer-to-peer central registry-biased Internet of Everything Protocol (P2PRioEP), serving as an application support registry for hybrid peer-to-peer IoT networks. The results obtained over a set period of time are presented and discussed.

d. Data Quality Assurance

Remote sensing is a major source of data, providing data for Earth observation and analysis from satellites, aircraft, and ground-based structures. In PA, a paradigm that emphasizes site-specific management, agricultural remote sensing is an essential technology. The authors of [124] explore the challenges and opportunities presented by agricultural remote sensing data, which share characteristics with general remote sensing data. The study provides an overview of remote sensing data resources, recent technological advances in remote sensing, big data management, and the processing and management of these data for PA. The paper proposes a bespoke Four-Layer, Twelve-Level remote sensing data management framework (FLTL), addressing the unique needs of PA. This FLTL framework, adapted from the Original Five-Layer, Fifteen-Level (FLFL) structure, serves as a comprehensive model for managing and applying agricultural remote sensing big data in PA and local agricultural studies.

In the study reported in [125], the potential of combining time series of crop yield monitoring data from multiple fields and years into a unified dataset for predictive modeling using ML approaches was explored. Using data from large farms in Western Australia, the study integrated crop yield monitoring data for wheat, barley, and canola over three seasons (2013, 2014, and 2015), covering a vast number of hectares each year. The data were processed, gathering spatial and temporal predictor variables, and aggregated to a spatial resolution of 100 m for yield modeling. Random forest models were used to predict crop yields at different times of the season, corresponding to pre-sowing, mid-season, and end-of-season conditions. The models demonstrated accurate predictions, with root mean square errors ranging from 0.36 to 0.42 t ha^−1^ and Lin’s concordance correlation coefficients from 0.89 to 0.92 at field resolution. Forecast accuracy improved as the season progressed, in line with the increased availability of in-season information, such as precipitation. The study highlights the generic applicability of the method to other agricultural systems with available yield monitoring data, suggesting avenues for future work to integrate additional data sources and refine spatial resolutions within fields.

## 4. Applications and Case Studies

The literature provides an in-depth exploration of the role of WSNs in agriculture, focusing on numerous crops to assess WSN applicability. The main objective is to use WSNs for crop monitoring, irrigation management, and the measurement of various environmental parameters crucial for better crop growth and quality. The researchers studied the impact of WSNs on eighteen different crops, ranging from broccoli, cotton, oranges, and orchids to sugarcane, grapes, peppers, potatoes, citrus, blueberries, corn, wheat, peaches, and more [35]. Importantly, WSNs are widely used to monitor crop growth in greenhouses. Another area of the research looks at the communication technologies adopted in WSNs for PA, with Bluetooth, ZigBee, Wi-Fi, and GPRS/GSM identified as the predominant options. Notably, WiMAX technology is currently excluded from PA applications for reasons of cost and size. ZigBee appears to be the most popular communications technology due to its wide transmission range and cost-effectiveness. GSM/GPRS comes in a close second, making it the second most widely used communication technology in PA via WSNs [35].

WSNs consist of interconnected wireless nodes equipped with radio transceivers, microcontrollers, sensors, and antennas to monitor environmental parameters. Sensors, measuring various factors such as soil statistics, crop characteristics, weather conditions, and resource requirements, transmit data to controllers, which then relay the information to the cloud or portable devices [126]. The agricultural sector, with its multifaceted demands, benefits from WSN technologies, facilitating real-time monitoring of crop behavior. Continued advances in WSN technologies have led to a reduction in the size and cost of sensors, enabling their widespread implementation in various sectors, including agriculture. Table 4 presents examples of sensors used in PA.

a. Precision Irrigation

The study reported in [147] focuses on solving the challenges of irrigation efficiency, water scarcity, and labor-intensive tasks in agriculture. It proposes an intelligent irrigation system aimed at contributing to PA. Integrating various sensors to measure water reservoir levels, humidity, temperature, soil moisture, and precipitation, as well as retrieving weather forecasts for temperature and precipitation, the system uses an algorithm programmed using Python 2.7 involving a relay control approach, given the complex decision-making process described. Using this algorithm, the smart irrigation system optimizes resource use, maximizing crop yields. In addition, the inclusion of a web portal improves accessibility for farmers by providing real-time information on water tank levels, water pump status, weather forecasts, and sensor measurements. This comprehensive approach aims to meet the challenges of water scarcity and time constraints associated with traditional irrigation methods in agriculture.

In another [148] study, the authors highlight the demand for an automated telemetry system for irrigation, aligned with market interests in energy-saving and cost-effective solutions for agriculture. The proposed system features a favorable architecture characterized by minimal energy consumption, low management costs, scalability, forecasting capabilities, diagnostic features, and easy expansion, indicating substantial technical impact and marketing potential. The main objective is to implement an integrated solution for automation and telemetry in PA, prioritizing energy efficiency and taking advantage of the latest technologies on the market. The system involves comprehensive monitoring of crucial crop parameters and introduces an irrigation control automation system designed to reduce energy consumption. Telemetry processes are facilitated by an ADCON telemetry station, measuring field parameters, and transmitting data to the SCADA system via an RTU (Remote Terminal Unit) and communication server. Connection to PCs requires OPC connections via a gateway. Automatic control and regulation are carried out by PLCs (Programmable Logic Controllers) using control algorithms and command elements, with communication facilitated via GSM modules for network simplification. Fuzzy control algorithms calculated in MATLAB are transmitted to a PLC for implementation, facilitating fuzzy control.

As part of the IoT, the SWAMP project addresses the imperative need for seamless integration of various technologies for smart and efficient water management in precision irrigation for agriculture. The authors of [149] describe pilot-based deployments of the SWAMP architecture, platform, and system in Brazil and Europe. The focus on replicability is evident, and scalability issues are addressed through performance analysis of FIWARE components. The Guaspari scenario, a simplified version of MATOPIBA, also highlights the adaptability of SWAMP architecture to different contexts. The Guaspari deployment includes a streamlined Fog Hub and a cloud-based IoT agent, optimizing the system’s robustness against failures while providing insight into the potential trade-offs between robustness and connectivity. The study contributes to the understanding of IoT-based smart water management, offering valuable insights for wider applications and addressing the challenges of scalability and adaptability in diverse agricultural contexts.

The authors of [150] present an intelligent irrigation system, AREThOU5A, which exploits advanced technologies, including the IoT and machine learning, to revolutionize traditional agricultural practices. The innovative aspect lies in the integration of 5G networks and Radio Frequency Energy Harvesting (RF EH) into the IoT platform. The AREThOU5A platform aims to efficiently manage irrigated water in PA by integrating data from a wireless sensor network and satellite information. In particular, the platform uses LPWAN, RF energy harvesting, and ML to advance sustainable agricultural practices. The study includes the fabrication and validation of a rectenna module for RF energy harvesting, demonstrating promising results in an outdoor environment. The AREThOU5A IoT platform encompasses several subsystems, such as measurement, routing, user interface, and server subsystems, each contributing to its complete functionality. This holistic approach integrates various technologies to optimize water use strategies for growers, presenting a notable contribution to the field of PA.

An irrigation support system based on automatic intelligent data mining for efficient irrigation management in agriculture is presented in [151]. The hybrid Convolutional Neural Support Vector Machine Classifier (CNSVMHC) is proposed as a key component for optimizing irrigation schedules using real-time soil Moisture Content (MC) data collected via a Wireless Sensor Network (WSN). The CNSVMHC, a hybrid of Convolutional Neural Network (CNN) and Support Vector Machines (SVM), facilitates a closed-loop control system, adjusting decision support schemes to account for approximation faults and local disturbances. The system aims to improve irrigation efficiency by taking into account soil, water, weather, and crop data, minimizing costs, maximizing crop productivity, and relying on real-time values rather than forecasts. The site-specific distributed irrigation system based on field sensors, combined with an intelligent Decision Support System (DSS), is highlighted for its potential to maximize harvest and quality while conserving water. The study incorporates robust estimation methodologies, including neural network algorithms, to rectify the farming system model, particularly in non-linear systems. The proposed approach uses a WSN environment for real-time monitoring and assessment of soil MC, collecting data from various sensors and storing it in a gateway node with Wi-Fi connectivity. The data collected in real time is then sent to a computer server for soil MC forecasting using CNSVMHC.

The paper [152] discusses the urgent need for innovative approaches in agriculture to address challenges posed by rapid climate change, population growth, and shrinking arable land. It emphasizes the pivotal role of IoT technologies, particularly smart sensors, in revolutionizing greenhouse farming. Within an IoT-enabled greenhouse environment, numerous sensors and actuators are strategically deployed to continuously monitor and detect environmental changes, enabling data collection for analytics and supervision. The paper outlines a mesh network architecture for the greenhouse IoT system, featuring a local host with a GSM 2.5G receiver and microcontroller units. It also delves into the fundamentals of smart greenhouses, addressing emergency concerns like plant disease detection and exploring the efficiency of discriminant functions versus neural networks. The critical factors controlled within the greenhouse, including humidity, temperature, CO_2_ levels, light, Electrical Conductivity (EC), pH, and dissolved oxygen, are monitored by corresponding sensors and adjusted by five types of actuators. These actuators aim to create the ideal atmospheric conditions, manage water conditions, and enhance lighting. The Decision Support System (DSS) serves as the central operating system, orchestrating all activities for optimal greenhouse management.

b. Fertilizer Management

The authors of [153] present a system for water and fertilizer monitoring based on soil conductivity thresholds, using a cost-effective wireless sensor network for data collection and transmission to a decision support system. Using soil Electrical Conductivity and changes in moisture content, the decision support system guides the application of water and fertilizer, improving the accuracy of the control system. Experimental results indicate a 10.89% reduction in fertilizer use on average compared with traditional water-fertilizer integration control systems, potentially saving 0.76 to 0.87 tons of fertilizer over the cotton growing season. The system involves monitoring nodes deployed on farmland, collecting soil and environmental parameters, processing the data via a microprocessor, and transmitting it to the central control unit. The decision support system checks data packets against predefined thresholds, such as soil EC values, and controls the pump start/stop signal according to the difference between soil EC detection values and fertilizer EC values, enabling more efficient application of water and fertilizer.

In [154], an IoT-based fertilizer recommendation system for smart agriculture is presented, leveraging IoT devices and sensors to collect relevant agricultural data. The proposed framework uses ML techniques to provide fertilizer recommendations based on precise quantities and optimal timing. The data acquisition phase involves collecting input data such as soil temperature, humidity, weather data for specific regions, and crop details. Feature selection is performed using the sequential floating forward selection algorithm, and multilinear regression is used for data classification. Its performance is compared with Random Forest, C4.5, and Naïve Bayes algorithms, with SFSS-MLR demonstrating superior accuracy, precision, recall, and F1 metrics, achieving an accuracy rate of 99.3%.

In [155], an innovative IoT approach to improving crop productivity through real-time monitoring of soil nitrate levels is presented. Traditionally, fertilization decisions are guided by paper-based methods and canopy greenness measurements, leading to suboptimal applications and environmental issues. In response, the proposed system uses ion-selective membrane sensors and digital soil moisture probes to directly measure soil nitrate levels. Integrated with IoT connectivity, in particular Lora WAN transceivers, the system facilitates real-time data transmission to a cloud-connected gateway for processing and storage. The implementation of this soil sensor system aims to improve nitrogen use efficiency, support informed fertilizer management decisions, and mitigate nitrogen losses to the environment, thus contributing to improved agricultural sustainability.

A study reported in [156] examines the potential of Diamond-Attenuated Total Internal Reflectance (D-ATR) Fourier Transform Infrared Spectroscopy (FTIR) as a soil nitrate (NO_3_^−^) sensor for the precise management of Nitrogen (N) fertilizers. The research uses two datasets: one from the field with 124 GPS-recorded soil samples collected from four agricultural fields, and another in the laboratory with five different soils enriched with varying amounts of KNO3. Using partial least squares regression, D-ATR-FTIR spectroscopy demonstrates promising results with R^2^ calibration values of 0.83 and 0.90 for the field and laboratory datasets, respectively. In addition, robust cross-validation tests yield R^2^ values of 0.65 and 0.83, demonstrating the potential of D-ATR-FTIR spectroscopy for rapid on-the-move determination of soil NO_3_^−^ concentrations in the field. The field tests involve samples from four agricultural fields in central Iowa, taking into account variations in moisture, texture, organic matter, and other properties. The study suggests that this spectroscopic approach could enhance precise applications of nitrogen fertilizers, improving efficiency and reducing environmental impacts.

Another study [157] aims to solve the problem of overfertilization in ornamental nursery production by investigating the effectiveness of two non-destructive sensors, Soil Plant Analysis Development (SPAD-502) and GreenSeeker^TM^, in assessing nutrient uptake by plant tissues. The study uses Florikan Top-Dress 12N-6P-8K fertilizer on Justicia brandegeana with variable increments to simulate different nitrogen rates. Parameters such as NDVI, SPAD readings, total leaf Carbon:Nitrogen (C:N), and soil leachate analysis are recorded over three months. The results indicate that a smaller quantity (20 g) of Controlled-Release Fertilizer (CRF) is as effective as larger quantities (30, 40, and 50 g) in supplying nitrogen to J. brandegeana. The use of SPAD and GreenSeeker^TM^ optical sensors demonstrates promising results for determining fertilizer requirements in ornamental plant production, offering a rapid, non-destructive approach to sustainable fertilizer management practices in the industry.

Nutrient and fertilizer sensors monitor nutrient concentrations in real time, enabling precise and efficient nutrient distribution [158]. They ensure that crops receive the right nutrients precisely at the right time, reducing waste and minimizing environmental impact [159]. All of these types of smart sensors play a crucial role in improving farming techniques, reducing resource use, and enhancing crop production [160]. In this context, the integration of these sensors into an interconnected system forms the foundation of PA, enabling an era in which data-driven information directs all aspects of farming, from sowing to harvesting.

c. Pest and Disease Monitoring

Plant diseases, induced by elements such as pests, pathogens, and climatic changes, represent substantial threats to ecosystems and human life. Innovative sensor technologies for real-time monitoring and prediction of plant health are imperative to counter potential crop losses resulting from various stresses. Wearable plant sensors, positioned directly on organs from leaves to stems, seek to assess plant health by analyzing biomarkers and microenvironmental variables. These sensors, classified as plant growth, physiology and microclimate sensors, multifunctional sensors, and chemical sensors, offer promising applications in PA [161].

A revolutionary approach to real-time plant monitoring is proposed by a study [162] based on the development of a bionic plant sensor. This wearable plant sensor, featuring a nanometer-thick Ag film and double V-shaped grooves, addresses the limitations of existing multifunctional sensors, such as high manufacturing costs and suboptimal performance. The bionic sensor demonstrates exceptional vibration sensitivity and is manufactured using a simple, scalable strategy, with a remarkably low production time of one hour and a cost of USD 1.3 per unit. In addition, the wireless functionality enables continuous, real-time monitoring of bamboo growth, revealing day and night growth trends in line with established intrusive reports. The results suggest that this wearable, cost-effective plant sensor shows considerable promise for advancing real-time plant growth detection in PA.

Another study [163] explores advanced agricultural techniques for optimizing crop growth and yield by leveraging IoT technology for comprehensive crop monitoring and data collection. Research highlights the importance of understanding environmental conditions, soil quality, water, and fertilizer requirements throughout plant growth phases to improve agricultural productivity. Using an IoT-enabled monitoring system, the study aims to detect crop diseases and assess the reproductive status of pathogenic pests. Data analysis carried out on samples collected throughout the entire crop growth cycle reveals valuable information on the correlation between crop yield, weather parameters, and insect reproduction. Using a fuzzy inference system, the knowledge base is built using these meteorological parameters, and a multi-objective evolutionary algorithm applies fuzzy rules to identify suitable crop windows with low pest reproduction conditions and maximum crop yield. The proposed approach, tested on rice and sugarcane crops in the agricultural fields of Gwalior, India, uses an IoT-enabled wireless sensor network (IEEE 802.15.4) to collect data on soil moisture, precipitation, temperature, etc. Based on fuzzy logic, this study helps identify optimal planting seasons through IoT application development services, enabling farmers to proactively prevent the development of pests and achieve optimal yields.

A study reported in [164] presents an IoT-based intelligent palm monitoring prototype designed to remotely monitor palms using smart agricultural sensors, with a particular focus on early detection of red palm weevil infestations. Leveraging an industrial-level IoT platform, the prototype enables users to interact with their palm plantations via web/mobile applications and receive early alerts of infestation. Selecting suitable sensors for weevil detection was a tedious process, involving the testing of seven types, including vibration, accelerometer, and sound sensors. Of these, accelerometer sensors, in particular the Grove 3-axis ± 16 g ultra-low-power BMA400 digital accelerometer, proved the most effective in providing a distinctive signature of red palm weevil activity. The study highlights the challenges faced in sensor selection and the importance of early detection for effective pest control in intelligent agricultural systems.

## 5. Challenges and Future Directions

Precision agriculture, enabled by the integration of intelligent sensors and data analysis, has made significant advances in the optimization of farming practices. However, today’s path of transformation presents several challenges and constraints. An important obstacle lies in the deployment of smart sensors, where elements such as calibration, maintenance, and sensor durability influence the reliability of the data collected. Achieving extensive interoperability between various sensor networks also represents a challenge, hindering the seamless integration of data into agricultural systems. In addition, concerns about data accuracy and security require careful attention to establish trust between stakeholders. Constraints include reliance on historical data for predictive analysis, often at the expense of real-time environmental variations. Another limitation is that environmental factors such as soil composition and topography can influence the effectiveness of smart sensors, resulting in variations in data accuracy. The need for sustainable energy sources in remote areas remains a critical constraint, impacting the continuous monitoring of agricultural parameters.

Looking ahead, PA holds promising developments that can respond to these challenges and reshape the landscape. The integration of IoT technologies promises to revolutionize data collection, offering real-time information on crop health, soil conditions, and environmental changes. The use of big data analytics will enable more sophisticated predictive modeling, enabling farmers to make informed decisions. In addition, AI will play a central role in automated decision-making processes, offering advanced solutions for crop management, disease detection, and resource optimization. Furthermore, the emerging trends also suggest the potential for ML algorithms to be adapted in response to continuous data streams, thereby enhancing the adaptability of precision farming systems. Despite persistent challenges, the future of precision farming is promising. Future advances, driven by IoT, big data, and AI, promise to overcome existing limitations and position PA at the heart of sustainable and efficient farming practices. As research progresses and technologies evolve, the synergy between smart sensors and advanced analytics will pave the way for a new era of PA, further enhancing worldwide food security and environmental sustainability.

## 6. Conclusions

In this review, we present a comprehensive exploration of the evolving landscape of PA, characterized by the integration of smart sensors and advanced technologies. The papers discussed highlight the transformative potential of PA in improving crop management, resource use, and overall agricultural sustainability.

The challenges and limitations highlighted in these studies, such as sensor deployment issues, data accuracy problems, and the need for sustainable energy sources, underline the complexity of implementing precision farming systems. However, the review also points to the promising future of this field, where emerging technologies like the IoT, big data analytics, and Artificial Intelligence are expected to play a significant role.

The integration of IoT is set to revolutionize data collection, providing real-time information on crop health, soil conditions, and environmental changes. The analysis of big data will enable more sophisticated predictive modeling, enabling farmers to make informed decisions. AI, with its ability to automate decision-making processes, provides advanced solutions for crop management, disease detection, and resource optimization.

The synergy between smart sensors and advanced analytics, as illustrated by the articles reviewed, is the key to overcoming current limitations and making precision agriculture the foundation for efficient, sustainable farming practices. As ongoing research develops and technologies continue to improve, an optimistic trajectory remains for PA, making a significant contribution to global food security and environmental sustainability.

## Figures and Tables

**Table 1:** Examples of the platforms available for Smart Agriculture.

IoT Platform	Application	Reference
Phytoprove (2019)	Develop tools for early, rapid, and non-destructive determination of the nitrogen and water supply status of plants.	https://phytoprove.com/ (accessed on 1 March 2024)
Telemetry2u	Provides an integrated hardware and software system for measuring integrated temperature and irrigation control.	https://telemetry2u.com/ (accessed on 1 March 2024)
Sensoterra	Provides an integrated hardware and software system for measuring soil moisture.	https://www.sensoterra.com/ (accessed on 1 March 2024)
Cropx (2013)	Offers an integrated hardware and software system designed for the precise measurement of electrical conductivity, soil moisture, and temperature.	https://cropx.com/ (accessed on 1 March 2024)
ONFARM	Easily accessible critical information can be viewed in the office or on the go through a user-friendly dashboard.	https://www.onfarm.co/ (accessed on 1 March 2024)
Phytech	Optimizing irrigation through data-driven insights for enhanced plant health and resource management.	https://www.phytech.com/ (accessed on 1 March 2024)
Semios (2020)	Tool for enhancing yields by providing real-time assessments and responses to insect activity, disease, and overall plant health conditions.	https://semios.com/ (accessed on 1 March 2024)

**Table 2:** Comparison of existing communication technologies.

Parameters	Standard	Frequency	Data Rate	Transmission Range	Energy Consumption	Cost	Limitations
Wi-Fi	IEEE 802.11 [59]	2.4 GHz and 5 GHz bands	11–50 and 150 Mbps	20–100 m	High	High	High power consumption, limited range compared to other long-range technologies, and susceptibility to interference.
ZigBee	Zigbee Alliance IEEE 802.15.4 [60]	2.4 GHz and 868/915 MHz bands	20, 40, and 250 kbps	10–100 m	Low	Low	Line-of-sight connectivity should exist between the sensor node and the coordinator node.
Long-Range (LoRa)	Lora Alliance IEEE 802.15.4 [61]	Unlicensed ISM bands 868/915 MHz	50 kbps	<30 km	Very low	High	Low data rates, not suitable for high-bandwidth applications, scalability of the network, and capacity for messages.
SigFox	IEEE 802.15.4 [62]	Unlicensed ISM bands 868/915/433 MHz	100 bps	10–40 km	Low	Medium	Very low data rates, limited uplink capabilities, and a low payload limit for transmitted messages.
RFID	ISO/IEC 14443, ISO/IEC 15693. [63]	25 kHz, 13.56 MHz, and 860–960 MHz	40 to 160 kbp/s	1–5 m	Low	Low	Short-range, limited data storage on passive RFID tags, and potential for signal reflection and interference.
Mobile communication	N/A	Licensed bands 900–1800 MHz	Up to 170 kbps	1–10 km	Medium	Medium	Relatively high power consumption, especially for mobile devices, and may not be cost-effective for certain IoT applications.
Bluetooth	IEEE 802.15.1 [64]	2.4 GHz ISM band	1 to 3 Mps	1 to 10 m	Low	Low	Moderate range, higher power consumption compared to low-power technologies, and potential interference in crowded areas.
NB-IoT	3GPP release 13 [65]	LTE frequency bands	200 kbps	11–0 Km	Medium	High	Limited data rates, not suitable for applications requiring high bandwidth, and potential latency in communication.

**Table 3:** Examples of Satellites Used for Precision Agriculture and their Applications.

Satellite Name	Application in PA
ECOSTRESS	Evapotranspiration (ET) [89]
TERRA-ASTER	Water management [90]
Sentinel-1A	Soil Moisture [91]
Sentinel-2	Crop management [92]
Terra/Aqua MODIS	Crop yield [91]
KOMPSAT-2	Crop yield [93]
RapidEye	Crop yield [94] Soil water [95]
GeoEye-1	Nutrient management [96]

**Table 4:** Examples of Sensors Used in the Precision Agriculture Domain.

Sensor Name	Parameters	Reference
SDI-12 Sensor	Soil Moisture	[127]
Hydra probe II soil sensor	Soil Moisture, Soil Temperature, Conductivity and Salinity level	[128]
ECH2O-5TE Sensor	Soil Moisture content levels, Soil Electric Conductivity, Soil Temperature, Soil Organic Content, Soil Texture, and Soil Bulk Density	[129]
ECH2O EC-5 Sensor	Soil Moisture, Soil Temperature, Soil Water	[130]
EC250 sensor	Soil Temperature, Soil Moisture, Salinity level, and Conductivity	[131]
SKU: SEN0193 sensor	Soil Moisture, Soil Temperature	[132]
VH-400 sensor	Soil Moisture sensor, Soil Temperature	[133]
DHT11/DHT22 sensor	Temperature and Humidity sensor	[134,135]
SHT31 Sensor	Temperature and Humidity sensor	[136]
SHT71, SHT75	Temperature and Humidity sensor	[137]
TAOS TSL262R	Luminosity sensor	[138]
TGS4161	CO_2_ sensor	[139]
S-THB-M002 sensor	Temperature and Humidity sensor	[140]
TCS3472 RGB sensor	Light sensor	[141]
Sense H2 sensor	Hydrogen, Plant Wetness, Plant Temperature, and CO_2_	[142]
MP406 sensor	Soil Moisture, Soil Temperature, Soil Dielectric Permittivity	[143]
Cl-340 photosynthesis	Plant Moisture, Photosynthesis, Plant Wetness, Hydrogen level, Plant Temperature and CO_2_	[128]
PTM-48A photosynthesis monitor	Plant Moisture, Photosynthesis, Plant Wetness, Plant Temperature and CO_2_	[144]
YSI 6025 and YSI 6131 chlorophyll sensors	Photosynthesis	[145]
HMP45C sensor	Air Humidity, Air Temperature, and Air Pressure	[146]

## Abbreviations

ADC	Analog-to-Digital Converter	MLR	Multiple Regression Analysis
AI	Artificial Intelligence	N	Nitrogen
ANFIS	Adaptive Periodic Threshold-sensitive Energy Efficient sensor Network protocol	NC	Network Coordinator
APTEEN	Adaptive Periodic Threshold-sensitive Energy Efficient sensor Network protocol	NCS	Neural Compute Stick
AWS	Amazon Web Services	NDVI	Normalized Difference Vegetation Index
BOP	Beacon Only Period	OASNDFA	Optimized Algorithm of Sensor Node Deployment for intelligent Agricultural monitoring
CDC	Complex Dielectric Constant	OSRS	Open-Source Remote Sensing
CNN	Convolutional Neural Network	PA	Precision Agriculture
CNSVMC	Convolutional Neural Support Vector Machine Classifier	PAF	Precision Agriculture and Farming
CRF	Controlled-Release Fertilizer	PAR	Photosynthetically Active Radiation
D-ATR	Diamond Attenuated Total Internal Reflectance	PCR	Principal Component Regression
DCTA	Dynamic Converge cast Tree Algorithm	PIS	Passive Infrared Sensor
DSS	Decision Support Systems	PLC	Programmable Logic Controller
EC	Electrical Conductivity	PLSR	Partial Least Squares Regression
ET	Evapotranspiration	PWM	Pulse-Width Modulation
FLFL	Five-Layer, Fifteen-Level	reNDVI	red-edge NDVI
FTIR	Fourier Transform Infrared Spectroscopy	RFEH	Radio Frequency Energy Harvesting
GCPs	Ground Control Points	RMSE	Root Mean Square Error
GD	Gradient Descent	RNN	Recurrent Neural Network
GHS	Greenhouse Gas	RTU	Remote Terminal Unit
G-IoT	Green IoT	SDGs	Sustainable Development Goals
GIS	Geographic Information Systems	SIS-PAF	Smart Irrigation System for Precision Agriculture and Farming
GNDVI	Green Normalized Difference Vegetation Index	SNR	Signal-To-Noise Ratio
GPR	Gaussian Process Regression	SPAD	Soil Plant Analysis Development
GPS	Global Positioning Systems	SSIM	Structural Similarity Index
GPU	Graphics Processing Unit	SVM	Support Vector Machines
IAS	Irrigation Advisory Services	TAK	Title, Abstract, and Keywords
IoE	Internet of Everything	TVWS	TV Whitespace
IoT	Internet of Things	UAVs	Unmanned Aerial Vehicles
IWUE	Irrigation Water Use Efficiency	UWB	Ultra-Wide Band
Lasso	Least absolute shrinkage and selection operator regression	VLRGD	Variable Learning Rate Gradient Descent
LCIS	Low-cost System for agricultural Irrigation Support	VWC	Volumetric Water Content
LDR	Light-Dependent Resistor	Wi-Fi	Wireless Fidelity
LIBS	Laser-Induced Breakdown Spectroscopy	W-Mod	Water balance simulation Modeling
LoRa	Long-Range	WS	Web Server
LPWA	Low-Power Wide-Area	WSAN	Wireless Sensor and Actuator Network
LTMN	Long Short-Term Memory	WSNs	Wireless Sensor Networks
MAPE	Mean Absolute Percentage Error	WUSN	Wireless Underground Sensor Networks
MC	Moisture Content	ZigBee	Zonal Intercommunication Global standard
ML	Machine Learning		
